# Evaluating maize phenotype dynamics under drought stress using terrestrial lidar

**DOI:** 10.1186/s13007-019-0396-x

**Published:** 2019-02-04

**Authors:** Yanjun Su, Fangfang Wu, Zurui Ao, Shichao Jin, Feng Qin, Boxin Liu, Shuxin Pang, Lingli Liu, Qinghua Guo

**Affiliations:** 1 State Key Laboratory of Vegetation and Environmental Change, Institute of Botany, Chinese Academy of Sciences, Beijing, 100093 China; 2 University of Chinese Academy of Sciences, Beijing, 100049 China; 3 Guangdong Key Laboratory for Urbanization and Geo-simulation, School of Geography and Planning, Sun Yat-sen University, Guangzhou, 510275 China; 4 College of Biological Sciences, China Agricultural University, Beijing, 100091 China

**Keywords:** Maize, Phenotype, Lidar, Drought stress

## Abstract

**Background:**

Maize (*Zea mays* L.) is the third most consumed grain in the world and improving maize yield is of great importance of the world food security, especially under global climate change and more frequent severe droughts. Due to the limitation of phenotyping methods, most current studies only focused on the responses of phenotypes on certain key growth stages. Although light detection and ranging (lidar) technology showed great potential in acquiring three-dimensional (3D) vegetation information, it has been rarely used in monitoring maize phenotype dynamics at an individual plant level.

**Results:**

In this study, we used a terrestrial laser scanner to collect lidar data at six growth stages for 20 maize varieties under drought stress. Three drought-related phenotypes, i.e., plant height, plant area index (PAI) and projected leaf area (PLA), were calculated from the lidar point clouds at the individual plant level. The results showed that terrestrial lidar data can be used to estimate plant height, PAI and PLA at an accuracy of 96%, 70% and 92%, respectively. All three phenotypes showed a pattern of first increasing and then decreasing during the growth period. The high drought tolerance group tended to keep lower plant height and PAI without losing PLA during the tasseling stage. Moreover, the high drought tolerance group inclined to have lower plant area density in the upper canopy than the low drought tolerance group.

**Conclusion:**

The results demonstrate the feasibility of using terrestrial lidar to monitor 3D maize phenotypes under drought stress in the field and may provide new insights on identifying the key phenotypes and growth stages influenced by drought stress.

## Background

In recent decades, the global climate change has brought more and more frequent heat-waves and severe droughts [17], which has become an explicit threat to the global food security [22]. Maize (*Zea mays* L.) is the third most consumed grain in the world and studying how to secure maize yield under drought stress is of great significance. Beyond improving the irrigation technology, cultivating maize varieties with high drought resistance potential is another effective way to reduce the influence of drought stress [47]. Crop phenotyping can provide crop trait estimations and help to identify the traits influenced by drought stress, which is a critical step for crop breeding [45, 47, 67].

Field-based method is the most commonly used for acquiring phenotype measurements currently [16] and has been widely used to assess the drought resistance of different crops [10, 64]. For example, Faroop et al. [24], Getnet et al. [26] and Xu et al. [66] found that drought stress can influence crop physiological metabolism, leaf size and yield based on field phenotype observations. Among various crop phenotypes, plant height and leaf area have been proved to be the key indictors related to drought stress [11, 23, 34, 52, 58, 71]. Maize plants have to reach a sufficient height to have enough photosynthate for yields, and drought stress can delay the plant development to influence yields [57]. The structure of crop leaves can influence the water and light use efficiency, which are important factors indicating the drought resistance [4, 38, 63]. The vertical structure of crop leaves is often represented by the leaf area density (LAD) and leaf area index (LAI) [33]. LAD is defined as the one-sided leaf area per unit of a horizontal layer volume [65], and the sum of LAD along the vertical profile is LAI [33]. The horizontal structure of crop leaves can be represented by the projected leaf area (PLA), which is defined as the percentage of the vertically projected canopy area to the total ground area. However, taking field measurements is very time-consuming and labor-intensive, and destructive harvesting methods are frequently used to obtain LAD and LAI. This limits most current studies only focusing on certain key growth stages, such as the tasseling stage and the ripening stage, which cannot reflect the cumulative impact of drought stress on crops through the growing period [14, 53, 71]. Therefore, it is of great significance to monitor the response of maize phenotypes to drought stress during the whole growing period using new crop phenotyping technology.

The development of near-surface remote sensing technology provides new opportunity for non-destructive, high-efficiency and high-resolution (both temporal and spatial) phenotyping. Vegetation indices derived from multispectral/hyperspectral imagery (e.g., normalized difference vegetation index and enhanced vegetation index) have been proven to be correlated to crop phenotypes, such as LAI, biomass, yield, and crop physiological processes [13, 30, 31, 48, 49]. Photogrammetry and computer vision technologies can be further used to estimate three-dimensional (3D) crop phenotypes [1, 7, 8, 15]. For example, Meyer and Davison [44] used images taken from two perpendicular directions to reconstruct 3D crop models and measure crop phenotypes (e.g. stem diameter and leaf angle) from the 3D models; Paproki et al. [50] successfully used 64 images taken from different angles to reconstruct 3D surface models of cotton plants; Duan et al. [19], Rovira-Más et al. [56] and Chen et al. [14] used the structure-from-motion method to derive 3D crop point cloud and measure crop phenotypes; Kise et al. [37] proved that the computer vision-based methods can be used to retrieve plant height at a centimeter-level accuracy. However, these imagery-based remote sensing methods are easily influenced by light conditions and cannot penetrate crop canopy, which limits their applications in field practices [42, 46].

Light detection and ranging (lidar), an active remote sensing technology, can provide accurate 3D information through measuring the time of flight of an emitted laser pulse between the sensor and the target. Besides, the focused short-wavelength laser pulse used by lidar sensors can effectively penetrate vegetation canopy and less influenced by the light condition [12, 21, 61]. Therefore, it has shown great potential for field-based high-throughput crop phenotyping [2, 3, 29, 32, 41, 51, 60, 62, 69]. However, lidar is still a relatively new technology to the field of crop phenotyping. Recently, more efforts have been spent on developing algorithms to automatically extract crop phenotypes from lidar data. For example, Jin et al. [35, 36] proposed methods combining deep learning algorithms with geometric principles to accurately extract 3D maize phenotypes (e.g., plant height, stem diameter, crown diameter, leaf area, leaf inclination angle, leaf length, and leaf width) from terrestrial lidar data. These studies further proved that lidar is an ideal tool for monitoring crop growth dynamics non-destructively in field practices. Nevertheless, to the best of our knowledge, no study has been conducted to explore the responses of 3D maize phenotypes to drought stress using lidar technology. The feasibility of lidar in monitoring maize phenotype dynamics and how maize phenotypes respond to drought stress cumulatively still need to be evaluated and analyzed.

The aim of this study is to evaluate the performance of lidar in monitoring time-series maize phenotypes in field practices and analyze the growth dynamics of different maize varieties under drought stress. Specifically, three questions were addressed. First, how accurate is lidar for maize phenotype extraction in field practices, and how do maize phenotypes change under drought stress during the whole growing period? Second, what maize phenotypes are associated with drought stress, and how can they indicate the occurrence and development of drought in 3D? Third, what are the key phenotypes that lead different maize varieties to have different drought resistance?

## Materials and methods

### Study site and field measurements

**Fig. 1:**
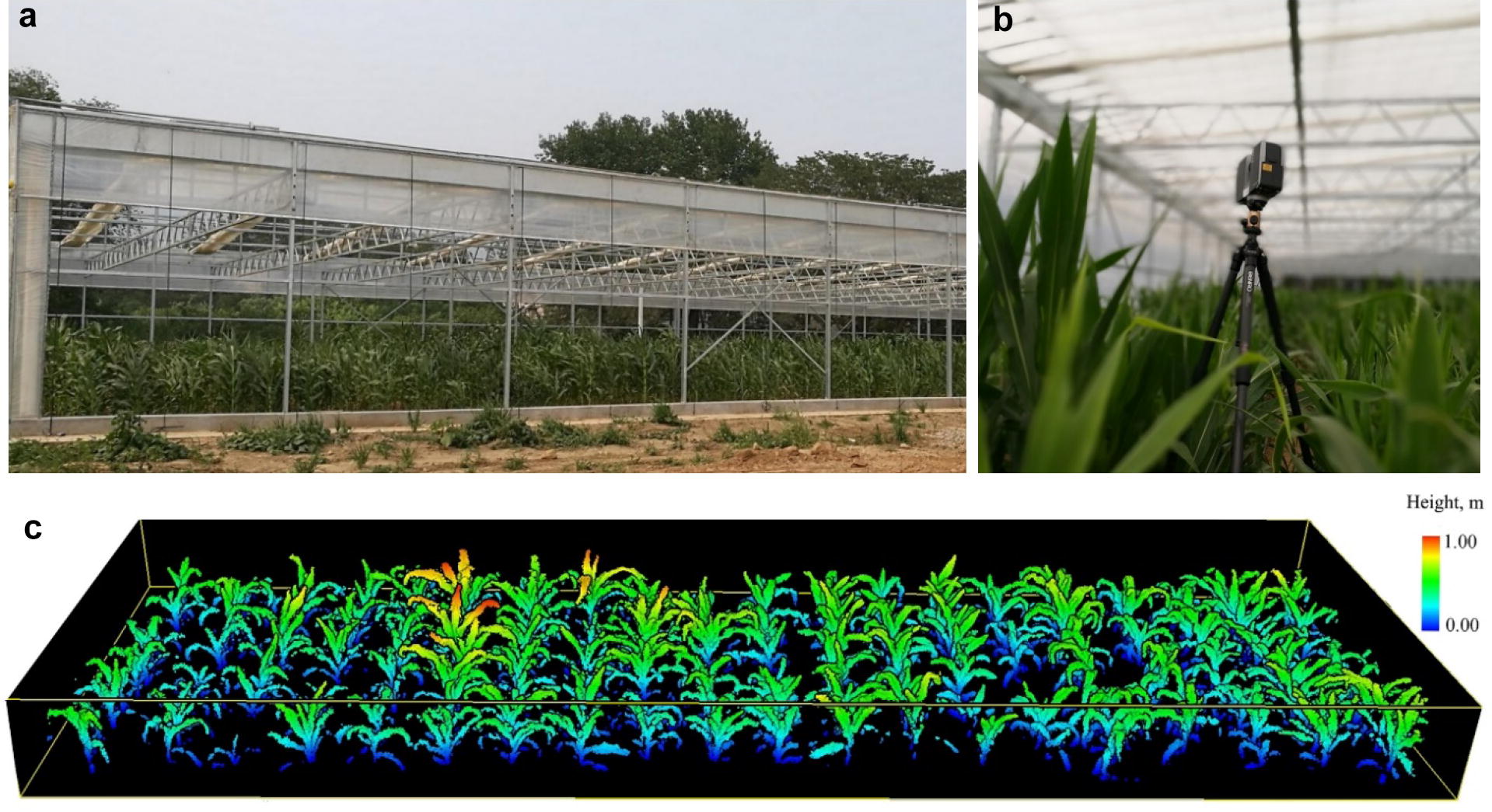
**a** The maize growth site with a rain shelter for simulating drought conditions; **b** the internal view of the study site and an illustration of the laser scanner setup for collecting lidar data; and **c** an example of the collected lidar point cloud on June 20th, 2016

The study site is located in the Institute of Botany, Chinese Academy of Sciences, Beijing, China (39°59′10″N, 116°12′21″E) with an area of 800 m^2^ (40 m × 20 m), and the soil type is yellow brown soil. To simulate a growth environment under drought stress, the study site was installed with a rain shelter. As can be seen in Fig. 1a, b, a layer of plastic film was installed at a height of 4 m to block natural rainfall. The rain shelter was opened all the time unless there were rainfalls. Moreover, a water-resistant barrier was installed below the ground to prevent water from surrounding soils penetrating to the study site.

To further reduce the influence of wind and edge effect, we sowed 20 maize varieties in the middle of the study site (10 m × 3 m) on May 10th, 2016, and we harvested them on September 20th, 2016. All maize individuals were planted in a regular grid. The distance between each column was 50 cm, and the distance between two adjacent plants along a column was 30 cm. Each column represented one maize variety with 10 individual plants (Fig. 1c). All maize varieties were watered during the first 20 days from sowing (before May 30th, 2016) to ensure the survival rate. The soil moisture was maintained at a level of higher than 30% (volumetric water content) during this stage. Since May 31st, 2016, all maize varieties were not watered anymore, and that day was counted as Day 0 (D0) under drought stress hereafter.

**Fig. 2:**
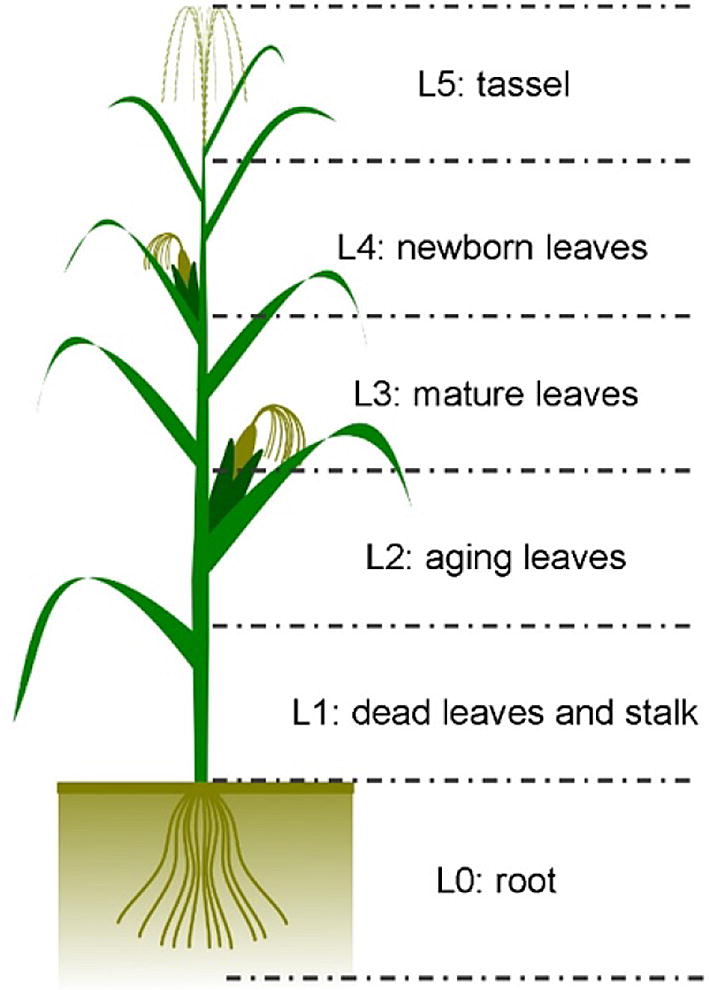
A demonstration of the division of maize vertical layers in this study. Note that the root layer (L0) was not included in the following analysis of this study

To collect ground truth measurements of maize phenotypes, 34 maize individuals were randomly chosen, and their plant height, plant area index (PAI) and PLA were manually measured. Their plant heights were measured with a staff at six key growth stages separately, covering from the early leaf emergence stage to the final mature stage (Table 1). A DJI Mavic Pro was used to capture an image right above each plant at a height of 5 m above the ground at the ripening stage D70. Each individual plant was then cropped out to calculate the PLA using the method proposed by Richardson et al. [55]. Moreover, each individual plant was divided into five height strata (Fig. 2). All leaves at each height layer of each individual plant were harvested separately (after the stage D95) and scanned using a Canon LiDE 220 scanner. If a leaf was intersected with two or more height layers, it was broken off from the thresholding height and each layer only harvested the part belonging to it. The scanned images were processed by the software of WinFOLIA to derive plant area density (PAD) at each height layer and therefore calculate PAI for each plant. Note that PAD and PAI were commonly used to replace LAD and LAI when leaves can be hardly separated from other organs [33].

**Table 1:** The six maize key growth stages used in this study and their corresponding dates

Date	Days since sowing	Days of drought stress^a^	Growth stage^b^
2016-06-20	40	D20	V6
2016-07-05	55	D35	V10–V11
2016-07-14	65	D45	VT
2016-07-29	80	D60	R1
2016-08-07	90	D70	R2–R3
2016-09-01	115	D95	R6

^a^Days of drought stress were counted since May 31st, 2016 (D0) when all maize plants were not watered anymore

^b^The growing stage was determined by the standard provided by Bondesio et al. [9]. V6 represents the stage that plants have 5 leaves, growth point is 20–25 mm below the ground, and cob and tassel is at initiation stage; V10–V11 represents the stage of cob development with around 10–11 leaves; VT represents the beginning of pollination stage; R1 represents the end of pollination stage; R2–R3 represents the stage of kernel development; R6 represents the end of mass grain stage and plants are ready for being harvested

To analyze the drought tolerance level of each maize variety, we planted a control group with the same 20 maize varieties in a field nearby the study site. Maize individuals of the control group were sowed and harvested in the same day as the group under drought stress and the same rules were used to manage them, except that they were watered all the time to keep the soil moisture higher than 30% (volumetric water content). After being harvested, the yields of all plants from both the control group and the group under drought stress were collected, dried, weighted and recorded. In this study, plant yields represent grain yields instead of biomass yields.

### Terrestrial lidar data collection and preprocessing

To cover the whole growing period, we collected six sets of terrestrial lidar data under drought stress at six key growth stages of maize (Table 1). A FARO Focus^3D^ X120 laser scanner in the high-resolution mode was used to acquire lidar data at five scanning positions surrounding the maize plants at each growth stage. The specification of the laser scanner is listed in Table 2. The five scanning positions were fixed for the lidar scans of all growth stages, and each scan was set up at a height of 1.5 m above the ground (Fig. 1b). To register the point clouds from different scanning positions, we put 10 target balls with a high reflectance in the scene, and at least four target balls were ensured that could be visually seen at each scanning position. The FARO SCENE 5.4.4 software was used to register the point clouds from different scanning positions for each growth stage, and the final registering error was around 2 mm on average.

**Table 2:** Specifications of the FARO Focus^3D^ X120 laser scanner used in this study

Field of view	Horizontal: 0°–360°
	Vertical: 30°–330°
Emission point density	976,000 points
Scan speed	122.000–922.000 Hz
Laser scan resolution	0.009°
Scanning accuracy	2 mm @ a 25 m distance
Scan distance	0.6–153.49 m
Laser wavelength	905 nm
Camera resolution	70 million pixels
Tilt sensor	± 5°
Scanner weight	4.9 kg

**Fig. 3:**
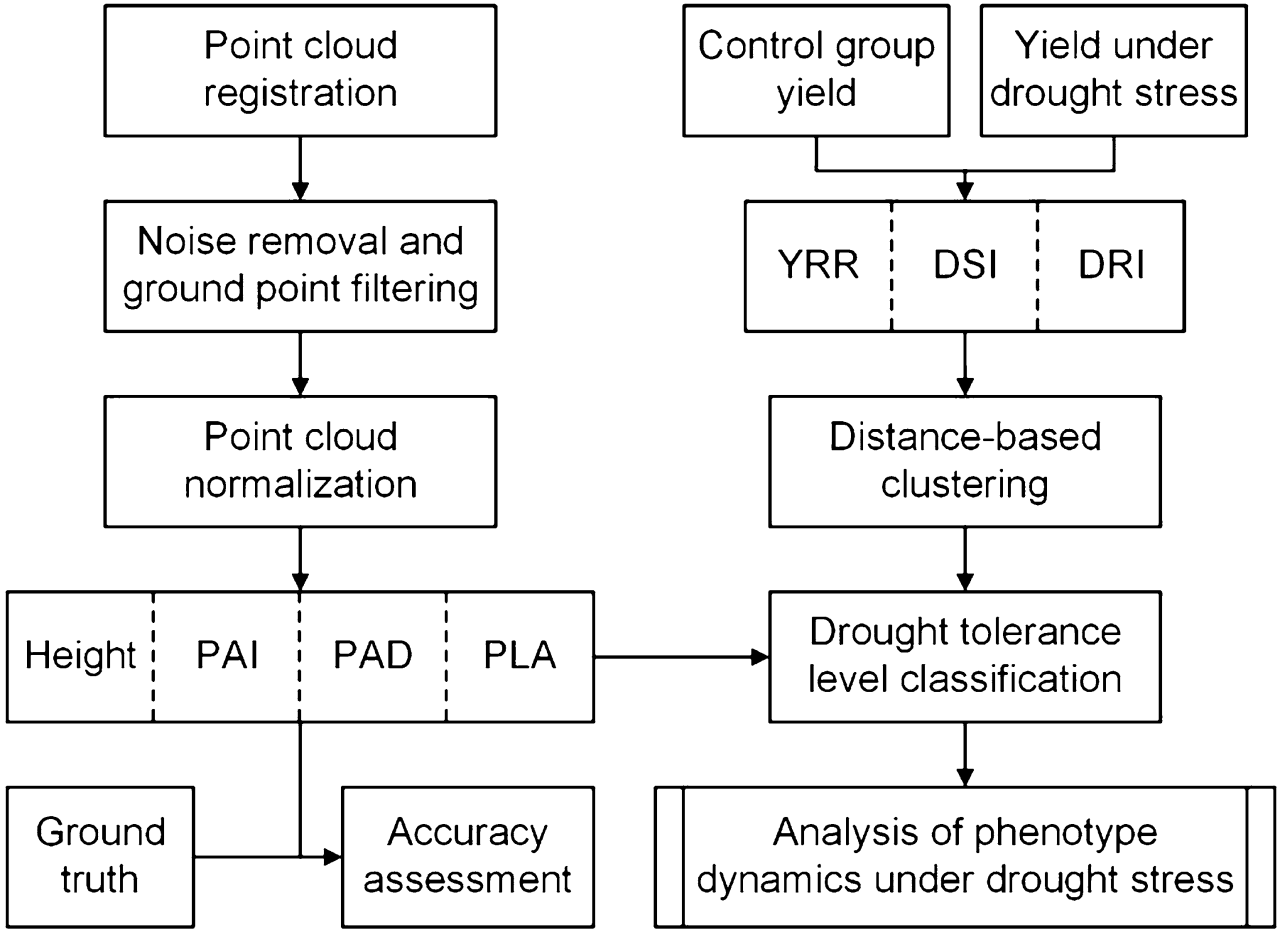
Scheme for processing the collected lidar point clouds and analyze the phenotype dynamics under drought stress. PAI, PAD, PLA, YRR, DSI and DRI represent plant area index, plant area density, projected leaf area, relative yield decrease, drought susceptibility index and drought resistance index, respectively

Noise points are inevitable in lidar data due to object occlusion, wind and so on. In this study, we used the outlier removal algorithm integrated in the Green Valley International LiDAR360 software to reduce noise points in the collected lidar data (Fig. 3). This algorithm identifies outliers based on the rule that whether the distance of a point to its surrounding neighbors is larger than *avg. *+ *n *× *std.* (where *avg.* and *std.* is the average distance of points to their surrounding neighbors, and *n* is a user-defined threshold). Then, the improved progressive triangulated irregular network densification filtering algorithm proposed by Zhao et al. [70] was used to classify ground points and non-ground points (i.e., vegetation points in this study) for the lidar data of each growth stage. A digital terrain model (DTM) in 5 cm resolution was calculated from the lidar ground returns using the ordinary kriging method [28]. The obtained DTM was used to normalize the lidar point cloud by subtracting the ground elevation from the original lidar elevation. Moreover, although the same data collection setting was used for all the six growth stages, the collected lidar point density still increased with the growth of maize plants because of the increase of environmental complexity. To make the lidar data of the six growth stages be comparable to each other, we resampled the lidar point cloud to make sure all lidar data have the same average point distance.

### Phenotype extraction from lidar data

It has been found that phenotypes related to maize plant height and leaf area are highly correlated to drought stress [11, 52, 71]. Therefore, in this study, we calculated the plant height, PAI, PAD and PLA for each maize individual from the lidar data of each growth stage for drought stress analysis. To derive these four parameters for each individual maize, we need to first identify and segment each individual plant from lidar point clouds. Because all maize individuals were planted in a regular grid with large intervals, we created a simple grid with a size of 50 cm × 30 cm and treated the points in each pixel as one maize individual.

The height of each plant was calculated as the maximum height from the ground in the corresponding pixel. PAI was calculated from the voxelized lidar data using the voxel-based canopy profiling method proposed by Hosoi and Omasa [33]. The point cloud at each growth stage was first voxelized with a given voxel size, and the attribute of each voxel was determined by whether there was at least one vegetation point in it. If there was one or more than one vegetation points in a voxel, its corresponding attribute was assigned as 1; otherwise, it was assigned as 0. Then, we divided a maize individual into five height strata, as shown in Fig. 2. The PAD of a height layer was calculated using the following equation,

1 $$PAD_{k} = \frac{{\cos \theta_{c} }}{{G(\theta_{c} )}} \times \frac{1}{\Delta H} \times \frac{{n_{l} (k)}}{{n_{l} (k) + n_{p} (k)}}$$

in which, $\theta_{c}$ represents the incident angle of a laser pulse, $n_{l} (k)$ and $n_{p} (k)$ represent the number of voxels with an attribute of 1 and 0 at the *k*th height layer, respectively, $\Delta H$ represents the height difference of each height layer, and $G(\theta_{c} )$ represents the extinction coefficient. Since voxel size has a great influence on the PAD estimation [29], we selected three maize individuals from the control group and repeatedly estimated their PAD values at each height layer using a voxel size varying from 1 to 12 mm with a step of 0.5 mm. The estimated PAD values were compared with field measurements to find the optimized voxel size for PAD estimation. Finally, the PAI of a plant individual was calculated as the sum of PAD from the five height layers, which can be described as,

2 $$PAI = \sum\limits_{k = 1}^{5} {PAD_{k} }$$

PLA is defined as the projected area of vegetation canopy on the ground. In this study, we first projected the lidar points of each maize individual to the X–Y plane. Then, the minimum point distance on the X–Y plane was used as the pixel size to rasterize the projected lidar points. Pixels with point(s) were marked as 1, and pixels without point were marked as 0. The proportion of pixels with a value of 1 to the total number of pixels of a maize individual on the X–Y plane was the PLA estimation.

The lidar-derived plant height, PAI and PLA estimations for the 34 independent maize samples were compared with field measurements. Two statistic measurements, i.e. coefficient of determination (*R*^*2*^) and root-mean-square error (RMSE) were calculated to assess the estimation accuracy.

3 $$R^{2} = 1 - \frac{{(n - 1)\sum\nolimits_{i = 1}^{n} {(x_{i} - \hat{x}_{i} )^{2} } }}{{(n - 2)\sum\nolimits_{i = 1}^{n} {(x_{i} - \bar{x})^{2} } }}$$

4 $$RMSE = \sqrt {\frac{{\sum\nolimits_{i = 1}^{n} {(x_{i} - \widehat{x}_{i} )^{2} } }}{n - 2}}$$

where $x_{i}$ is the ground truth measurement, $\hat{x}_{i}$ is the lidar-derived estimation, $\bar{x}$ is the average lidar-derived estimation, and *n* is the number of validation samples.

### Analysis of the influence of drought stress on maize phenotypes

#### Classification of drought tolerance level

Many drought tolerance indices have been proposed to evaluate crop drought resistance capability. However, most of these indices have their own limitations, and cannot be used alone to classify drought tolerance level [14]. In this study, to avoid the limitations of single drought tolerance indices, a distance-based clustering algorithm was used to classify drought tolerance level from three commonly-used drought tolerance indices, i.e., yield reduction rate (YRR), drought susceptibility index (DSI) and drought resistance index (DRI). They were calculated from the field grain yield measurements using the following equations [6, 25, 40],

5 $$YRR = \frac{{Y_{m} - Y_{a} }}{{Y_{m} }}$$

6 $$DSI = \frac{{1 - Y_{a} /Y_{m} }}{{1 - Y{}_{A}/Y_{M} }}$$

7 $$DRI = \frac{{(Y_{a} )^{2} }}{{Y_{m} }} \times \frac{{Y_{M} }}{{(Y_{A} )^{2} }}$$

where *Y*_*a*_ represents the yield of a maize variety under drought stress, *Y*_*m*_ represents the corresponding yield of the control group, *Y*_*A*_ represents the average yield of all maize varieties under drought stress, and *Y*_*M*_ represents the average yield of all maize varieties of the control group. YRR is a direct measurement of yield decrease but cannot evaluate the sensitivity under different stress severities [39]. DSI and DRI considers the stress severity in their calculations, but they might be problematic to use under sever environmental stresses [43]. The distance-based clustering function integrated in the SPSS (Statistical Product and Service Solutions) software was therefore used to classify the maize varieties into three groups (i.e., high drought tolerance, medium drought tolerance, and low drought tolerance) [14]. Note that among the 20 maize varieties, three of them were not included in the drought stress analysis due to the incomplete samples in the group under drought stress (certain maize individuals died during the growth period).

#### Analysis of maize phenotype dynamics under drought stress

The average plant height, PAI and PLA and the corresponding standard deviations of maize varieties with the same drought tolerance level were calculated at each growth stage, and the change rates of each parameter compared to the previous stage were calculated. These statistics were used to analyze the change dynamics of phenotypes with different drought tolerance levels. Moreover, the statistical test was used to evaluate whether the differences in plant height, PAI and PLA were significant among different growth stages for each drought tolerance level. The null hypothesis was that there was no difference between the values of a phenotype from two growth stages. Besides, we further calculated the average PAD at each height layer for maize varieties with the same drought tolerance level. The time-series vertical PAD profiles from maize varieties with different drought tolerance levels were compared to analyze the responses of maize vertical structures to drought stress.

## Results

### Lidar-derived maize phenotypes

**Fig. 4:**
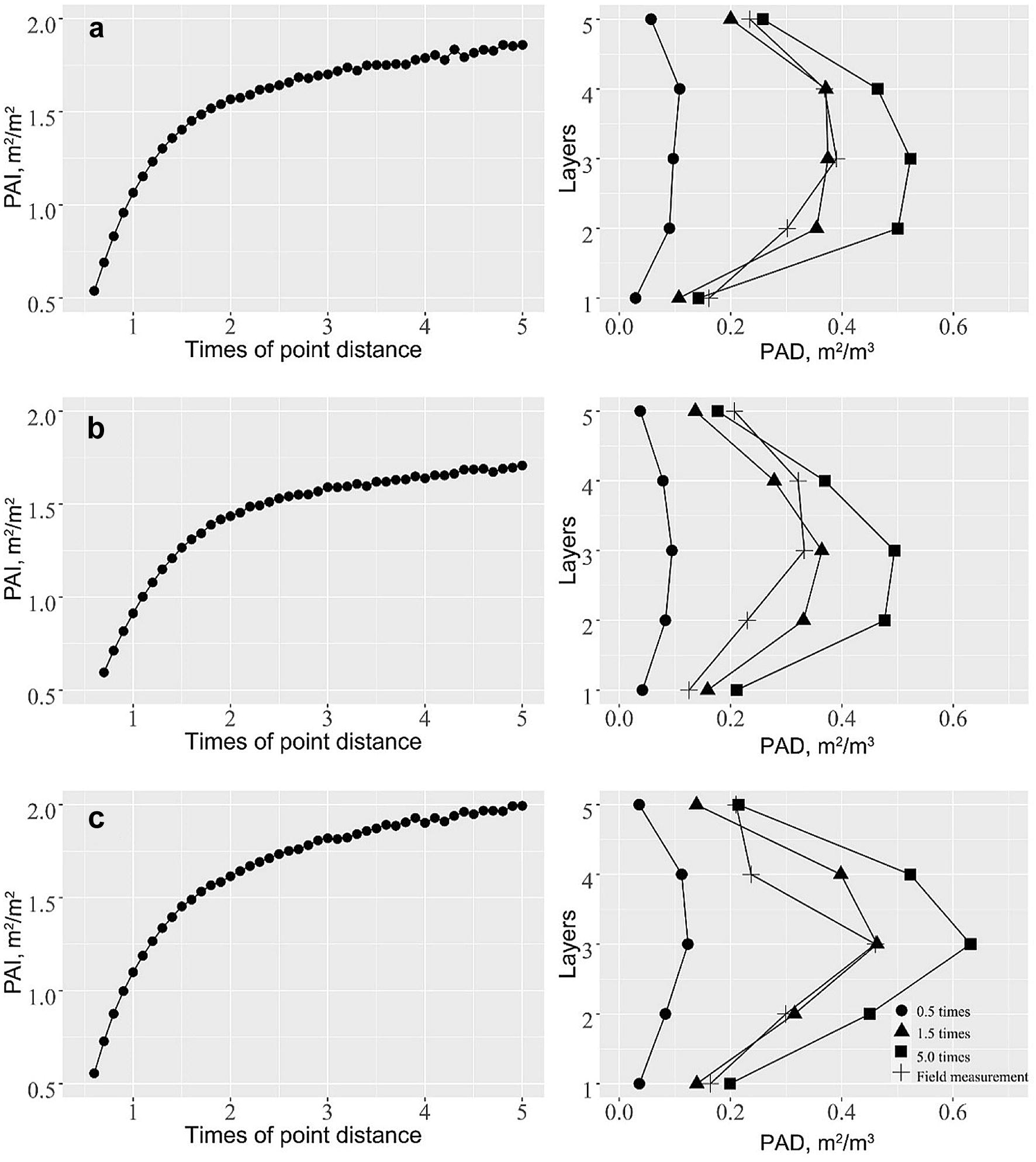
The influence of voxel size on the estimation of PAI (left column) and PAD at different height strata (right column). Each row represents a selected maize individual at the final growth stage. The PAD estimated from ground truth was compared with the lidar-derived estimations at different height strata on the right column. The five height layers correspond to the same five layers in Fig. 2, and the 0.5 times, 1.5 times and 5.0 times represent using a voxel size of the corresponding times of average point distance to estimate PAD from lidar data

The influence of voxel size on the PAI estimation from lidar is shown in Fig. 4. As can be seen, voxel size had a significant influence on the PAI estimation for all three testing maize individuals. With the increase of voxel size, PAI estimation first increased rapidly and then stayed relative stable after voxel size reaching a certain size. If the voxel size was too small, the voxel-based method underestimated the PAI; and if the voxel size was too big, the voxel-based method overestimated the PAI. In this study, we found that when the voxel size was set to 1.5 times of the average point distance, the estimated PAD at each height layer was close to the field measurements, and the final PAI reached a relative high accuracy as well. Therefore, a voxel size of 1.5 times of the average point distance of each maize point cloud was used to estimate the PAI of all maize individuals at each growth stage.

Table 3 shows the statistics of plant height, PAI and PLA for all maize individuals at each growth stage. Plant height, PAI, and PLA all reached their peaks at the growth stage of D60. The maximum plant height, PAI, and PLA can be three to four times higher than those at the beginning stage under drought stress. From D60 to D95, the average plant height, PAI and PLA decreased by 8%, 40%, and 20%, respectively. Moreover, the variations in plant height and PAI increased with the growth of maize plants. The standard deviations of plant height and PAI for the last three growth stages (i.e., D60, D70, and D95) were around three times higher than those of the growth stage D20. The variation of PLA stayed relatively stable during the growth period, and the smallest standard deviations appeared in the stage of D45. The proportion of standard deviation to average plant height was the lowest among the three phenotypes, which was only around 15% on average.

**Table 3:** Statistics of the lidar-derived plant height, PAI and PLA for all maize individuals at each growth stage

Growth stage	Height (m)^a^				PAI (m^2^/m^2^)^a^				PLA (m^2^/m^2^)^a^			
	Min	Max	Avg	Std	Min	Max	Avg	Std	Min	Max	Avg	Std
D20	0.41	0.70	0.55	0.09	0.24	1.31	0.65	0.29	0.09	0.18	0.13	0.03
D35	0.67	1.32	1.00	0.16	0.68	2.00	1.18	0.36	0.14	0.19	0.17	0.02
D45	1.20	1.83	1.42	0.17	0.82	2.85	1.60	0.52	0.16	0.21	0.18	0.01
D60	1.34	2.35	1.80	0.28	1.04	4.02	2.09	0.62	0.16	0.23	0.20	0.02
D70	1.31	2.28	1.76	0.28	0.31	2.85	1.70	0.82	0.12	0.20	0.18	0.03
D95	1.26	2.08	1.66	0.27	0.29	2.66	1.26	0.64	0.11	0.19	0.16	0.03

^a^Min, Max, Avg and Std represent the minimum, maximum, average and standard deviation of the corresponding phenotype of all plant individuals at each growth stage, respectively

**Fig. 5:**
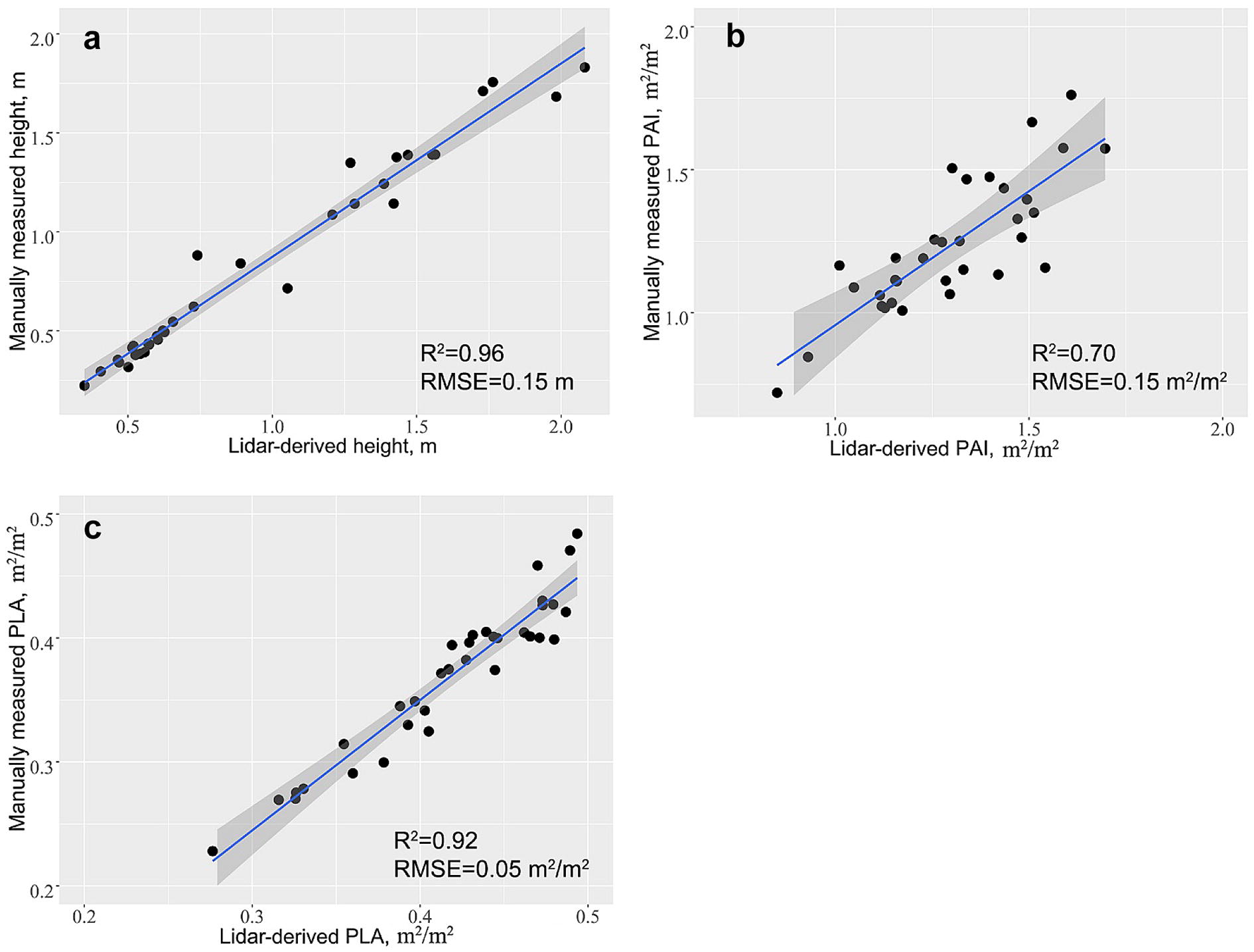
The comparison between the field-measured **a** plant height, **b** PAI and **c** PLA and the corresponding lidar-derived estimations

The estimated phenotypes were evaluated using field measurements of the 34 independent maize samples. Overall, all three lidar-derived phenotypes showed good agreements with field measurements (Fig. 5). Plant height had the highest estimation accuracy among the three phenotypes (*R*^2^ = 0.96, RMSE = 0.15 m) (Fig. 5a). Lidar-derived PLA showed a very high estimation accuracy as well with a *R*^2^ of 0.92 and a RMSE of 0.05 m^2^/m^2^ (Fig. 5b). Lidar-estimated PAI had the lowest accuracy among the three phenotypes with a *R*^2^ of 0.70 and a RMSE of 0.15 m^2^/m^2^ (Fig. 5c).

### Classification of drought tolerance level

**Fig. 6:**
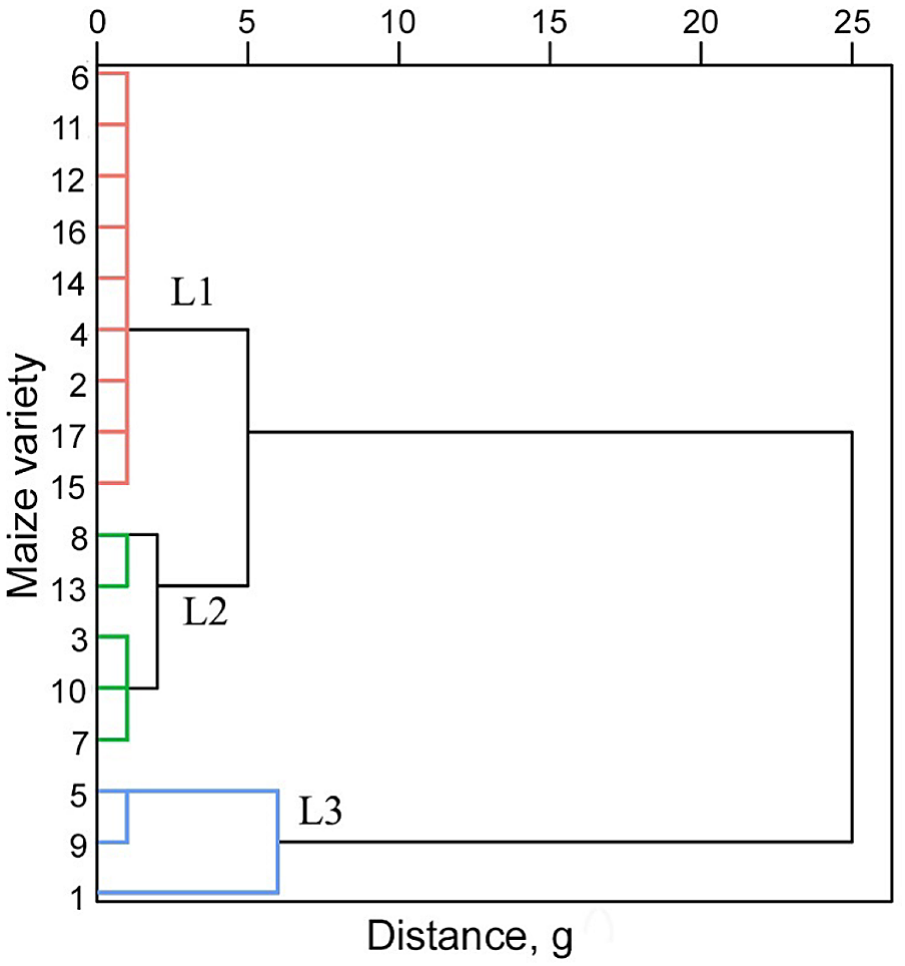
The distance-based clustering analysis for the maize drought tolerance level classification. L1, L2 and L3 represent the low drought tolerance level, medium drought tolerance level and high drought tolerance level, respectively

Based on the distance-based clustering analysis results, nine of the 17 maize varieties were classified as low drought tolerance (L1), five were classified as medium drought tolerance (L2), and three were classified as high drought tolerance (L3) (Fig. 6). The yield of nine maize varieties with a low drought tolerance decreased by 85% on average, and certain individuals totally failed during the growth period (i.e., producing no yield at all). The yield of five maize varieties with a medium drought tolerance decreased by 48% on average, and no individuals failed during the growth period. The yield of three maize varieties with a high drought tolerance decreased only by 27%, and the statistical test results showed that the yield of these three maize varieties had no significant difference with the control group (*p *> 0.05).

### Maize phenotype dynamics under drought stress

**Fig. 7:**
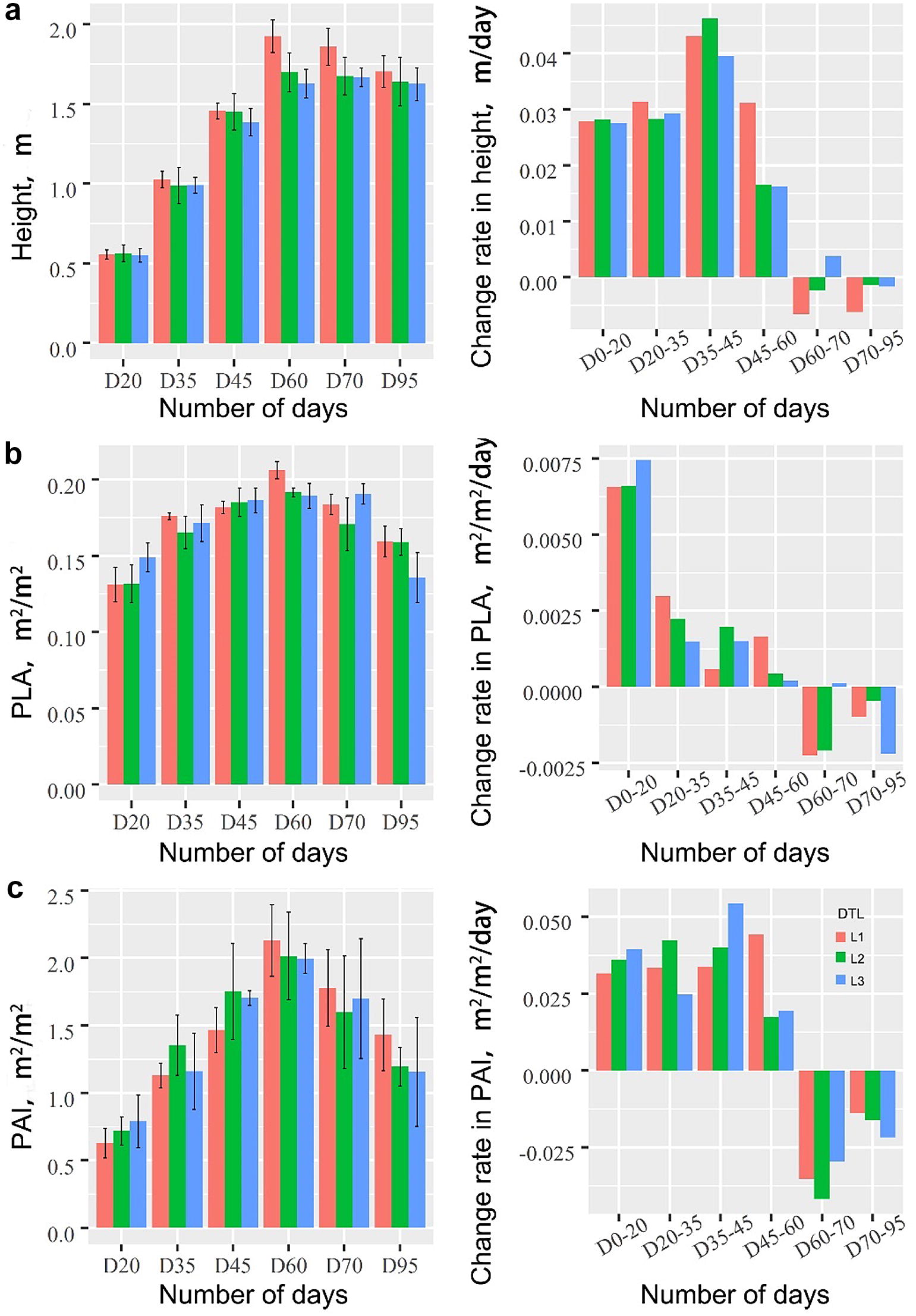
The growth dynamics of **a** plant height, **b** PAI and **c** PLA across the whole growth period (the right column), and the change rate of the corresponding parameter of each growth stage compared to its previous growth stage (the left column). Note that DTL represents the L1, L2 and L3 drought tolerance levels in Fig. 6

**Fig. 8:**
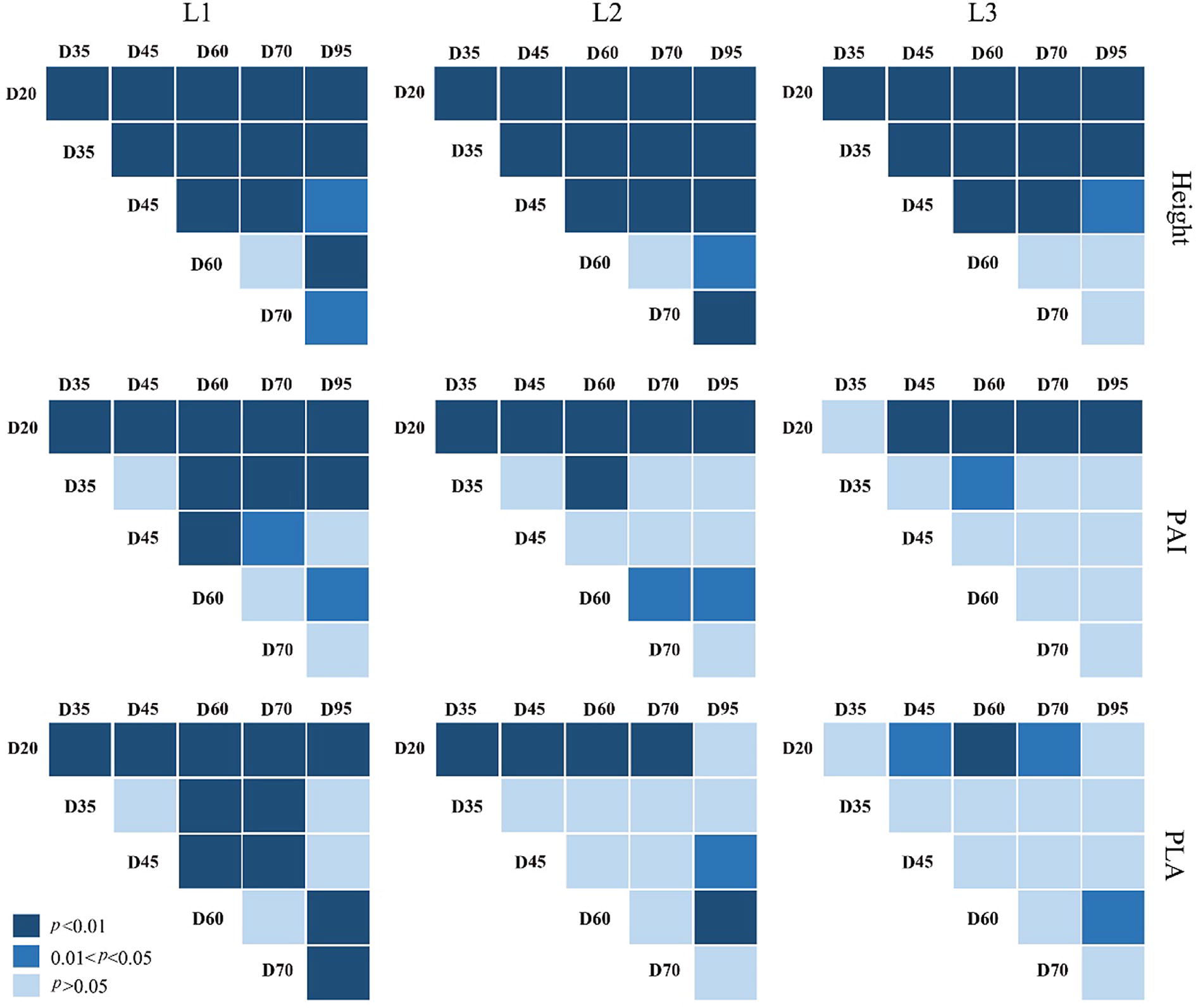
Statistic tests between phenotypes of one growth stage and other growth stages. L1, L2 and L3 represents the three drought tolerance levels in Fig. 6

The plant height of three drought tolerance groups all increased first and then decreased as the plant growth, and the height growth rate followed the same pattern (Fig. 7a). Before D20, the height differences among the three drought groups were the smallest, and the height growth rates were similar as well. From D20 to D45, maize individuals of all three drought tolerance groups increased significantly in plant height (*p *< 0.01), but the differences in plant height among three drought tolerance groups became larger (Figs. 7a, 8). From D45 to D60, the low drought tolerance group still kept a relative high growth rate in plant height, but the growth rates for the medium and high drought tolerance groups dropped rapidly. From D60 to D70, all three drought tolerance groups had no significant change in plant height (*p *> 0.05) (Fig. 8). After D70, the plant height of all three drought tolerance groups began to decrease, and the high drought tolerance group had the smallest drop in plant height. The statistic test result showed that the high drought tolerance group was the only group having an insignificant change in plant height among the three groups during this stage (*p *> 0.05) (Fig. 8).

The PAI of three drought tolerance groups followed a similar changing pattern as the plant height across the growth period, which increased first and then decreased (Fig. 7b). Before D20, the PAI values of three drought levels were close to each other, and the medium drought tolerance group had a relatively higher PAI than the other two groups. From D20 to D45, all three drought tolerance groups still had significant increases in PAI (*p *< 0.01), but the increase speed became much smaller (Figs. 7b, 8). The PAI of the medium drought tolerance group remained the highest among the three groups. From D45 to D60, the low drought tolerance group and high drought tolerance group still kept a relatively high PAI growth rate, but the PAI growth rate of the medium drought tolerance group began to decrease significantly. The low drought tolerance group replaced the medium drought tolerance group to have the highest PAI among the three groups, and it was also the only group having a significant change in PAI at this period (*p *< 0.05) (Figs. 7b, 8). From D60 to D95, the PAI of all three groups began to decrease, and the high drought tolerance group had the smallest change magnitude. The high drought tolerance group was also the only group having an insignificant change in PAI during these stages (*p *> 0.05) (Fig. 8).

The PLA of all three groups also followed the pattern of increasing first and then decreasing (Fig. 7c). Before D20, the PLA of all three groups increased rapidly. The PLA growth rate during this stage was the highest among all growth stages. Among three drought tolerance groups, the medium and high drought tolerance groups had a slightly higher PLA growth rate than the low drought tolerance group. From D20 to D60, all three drought tolerance groups still had continuous increases in PLA, but the increase speed became much slower. The low drought tolerance group was the only group having a significant change in PLA during this period (*p *< 0.01) (Fig. 8). All three drought tolerance groups had the highest PLA at the stage of D60, and the highest PLA values were close to each other. From D60 to D95, the PLA of all three groups began to have significant decreases (*p *< 0.05) (Fig. 7c, 8). The medium drought tolerance group had a relatively larger decreasing speed than the other two groups, and its PLA value at the final stage was the smallest among all three groups.

### PAD vertical profile dynamics under drought stress

**Fig. 9:**
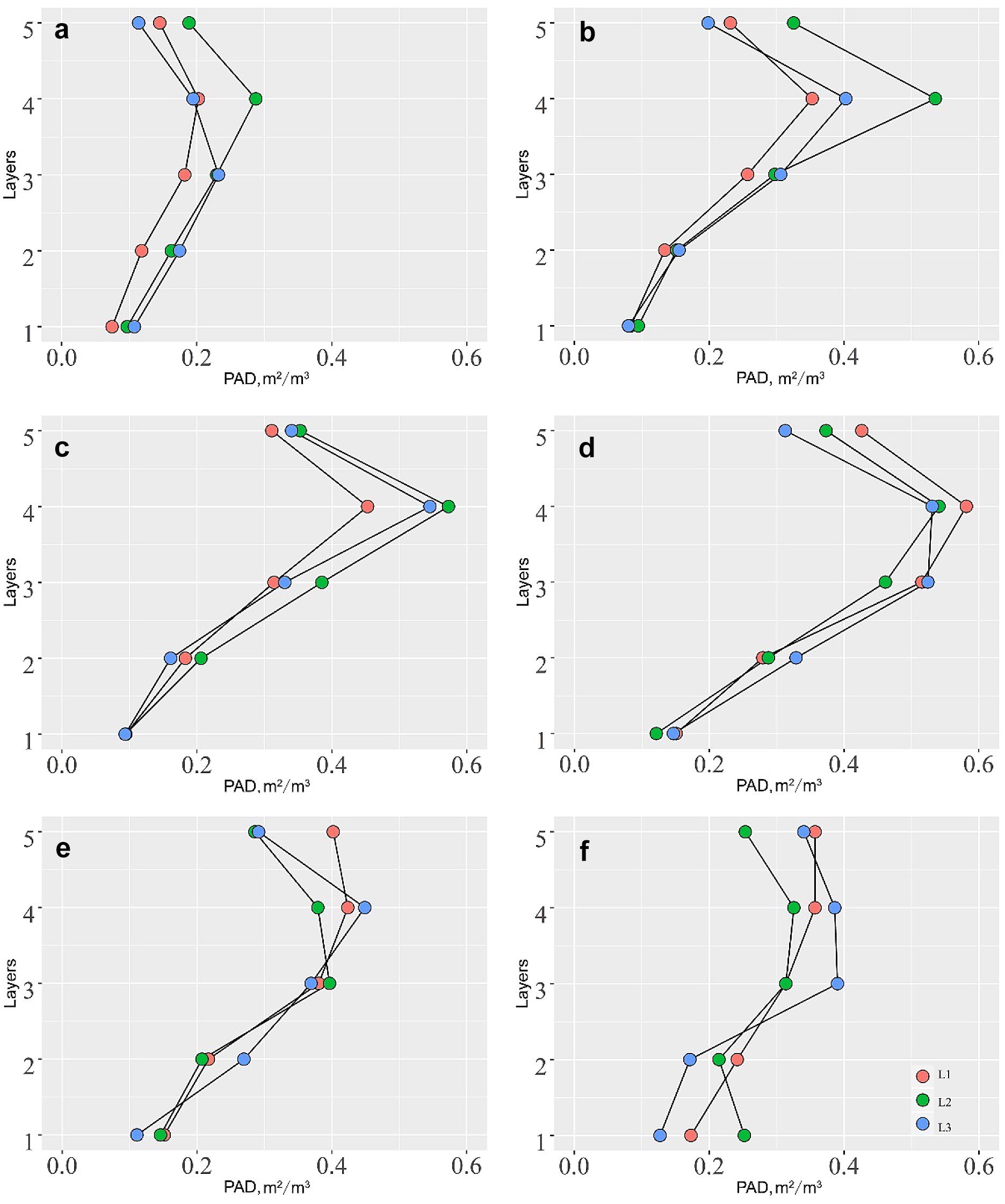
The PAD vertical distribution at different height strata of the growth stage **a** D20, **b** D35, **c** D45, **d** D60, **e** D70, and **f** D95. The five height layers correspond to the five layers in Fig. 2, and L1, L2 and L3 represent the corresponding drought tolerance group in Fig. 6

The PAD estimations at different height strata across the whole growth period were used to evaluate the vertical structure dynamics of maize varieties under drought stress (Fig. 9). From the seedling stage to D20, the upper level canopy for the medium drought tolerance group grew the fastest among the three groups, and the lower canopy for the low drought tolerance group grew the slowest (Fig. 9a). At the stage of D35, the upper canopy PAD for the medium drought tolerance group remained the highest, and the lower canopy PAD became close to each other for the three groups (Fig. 9b). At the stage of D45, the upper canopy of the high drought tolerance group grew quickly and became close to the medium drought tolerance group (Fig. 9c). The lower canopy of all three groups remained close to each other. At the stage of D60, the upper canopy for the medium and high drought tolerance groups remained relatively unchanged compared to the previous stage, but that for the low drought tolerance group continued to grow (Fig. 9d). The upper level PAD for the low drought tolerance group became the highest among all three groups at this stage. At the stage of D70, both the upper and lower canopy PAD began to decrease for all three groups, but only the shape of the PAD profile for the high drought tolerance group stayed relatively stable (Fig. 9e). The PAD for the most top layer of the low drought tolerance group had no significant changes, but that for the second top layer decreased significantly. As to the medium drought tolerance group, the third top layer had the smallest decrease in PAD which made it be the layer with the highest PAD. At the stage of D95, the PAD of all layers for all three groups continued to decrease, and the vertical structure profiles became more random (Fig. 9f).

## Discussion

### Sensitivity of maize phenotypes to drought stress

All phenotypes showed quick increases in the early growth stages and decreases in the final two growth stages. The decrease of plant height in the final two stages was caused by the fact that the loss of water in the ripening stages made the tassel branches could be easily broken [20]. The decreases of PLA and PAI in the final two stages were possibly caused by the fact that the loss of water in the ripening stages resulted in the rolling of leaves [20]. Since the broken of tassel branches was mostly random in the last two growth stages, but the rolling of leaves was systematic, the relative change of the plant height was the smallest compared to the relative decreases of PLA and PAI (Fig. 7).

**Fig. 10:**
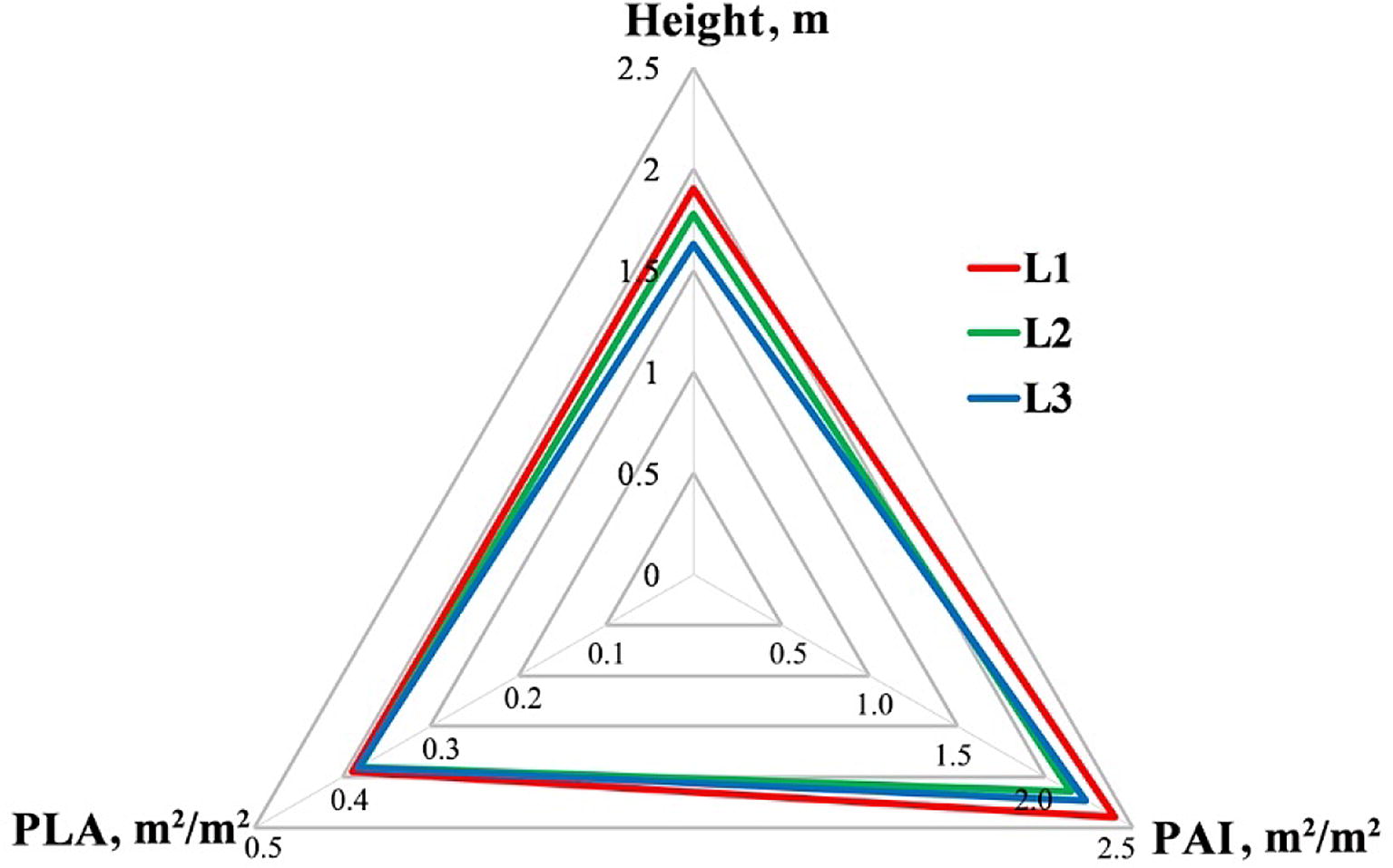
The distribution of average plant height, PAI and PLA of maize varieties with different drought tolerance levels at the growth stage of D60. L1, L2 and L3 represent the corresponding drought tolerance group in Fig. 6

The tasseling stage (D60) is the key maize growth stage which has the highest demand of moisture [18]. Therefore, it is the most sensitive stage of maize to drought stress. Figure 10 demonstrated the comparison of the three phenotypes of each drought tolerance group at the stage of D60. As can be seen, compared to plants with low drought tolerance, plants with high drought tolerance tended to keep a lower plant height and PAI. Lower plant height and PAI could reduce the transpiration and therefore reduce the demand for moisture during drought stress at the key growth stage [68]. Meanwhile, the PLA of maize plants with high drought tolerance stayed close to that of plants with low drought tolerance, which could help to ensure their light use efficiency for photosynthesis. The combining effect of these three phenotypes might be one of the reasons leading the high drought tolerance group to have higher yields.

From the 3D view, the PAI decrease at the key growth stage of D60 for the high drought tolerance group was caused by the relatively small PAD at the upper two height layers. As can be seen from Fig. 9d, the PAD of the upper two canopy layers become the lowest for the high drought tolerance group, while that of the lower canopy layers was close to each other. Although the upper levels of the high drought tolerance group had a similar number of leaves as the low drought tolerance group, the size of individual leaf at the upper levels of the high drought tolerance group was around 20% smaller than that of the low drought tolerance group. Zhang et al. [68] found that the transpiration rate and stomatal conductance of maize lower canopy in northern China was smaller than those of higher maize canopy due to the shading effect. Therefore, reducing the upper canopy PAD might be more efficient for maize plants to reach the goal of reducing water demand [54].

**Fig. 11:**
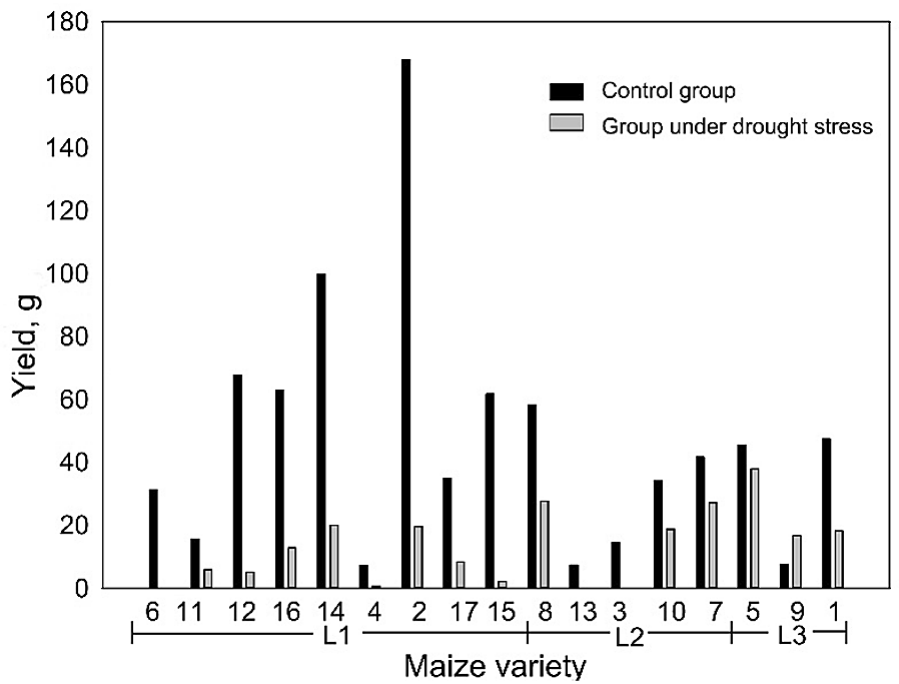
The comparison of average yield of each maize variety in the control group and in the group under drought stress. L1, L2 and L3 represent the corresponding drought tolerance group in Fig. 6

Considering the changing patterns of plant height, PLA and PAI of different drought tolerance groups across the growth period, the combination of low plant height and low PAI (especially at the upper level canopy) at the tasseling stages might be a good indicator to identify maize varieties with high drought tolerance level and predict the maize yield under drought stress. However, in this study, the yield of each individual maize had weak correlations with all three phenotypes (*R*^2^ < 0.3). The maize varieties selected in this study had different yield potentials. As can be seen in Fig. 11, although certain maize varieties fell in the group of low drought tolerance (e.g., No. 2 variety in L1), their corresponding yields were still higher than certain maize varieties of high drought tolerance group. To develop of a robust model for predicting maize yield from phenotypes at different growth stages, more strict control experiment on maize varieties and environmental conditions needs to be conducted in the future.

### The potential of lidar in field-based phenotyping practices

This study showed that lidar can provide accurate estimations of plant height and PLA. Although the estimation accuracy of PAI was relatively low compared to the other two phenotypes, the estimation accuracy still reached 70% and the RMSE only counted for around 10% of the average PAI value. The relatively low accuracy of PAI estimations might be caused by the following two reasons. First, there was a mismatch between field-based PAI measurements and lidar-derived PAI estimations. The stem, tassel and leaf sheath were very hard to be scanned, and it was difficult to break off leaves at the exact height threshold in the field if a leaf intersected with two height layers, which could possibly bring errors to the field-based PAI measurements. Second, some leaves of one maize plant might grow into the cubic space of another plant, and some maize point clouds from the individual maize segmentation step were incomplete because of the occlusion of leaves, which might bring uncertainty in the lidar-derived estimations. Recently, Jin et al. [35] proved that the deep learning technique can reach an accuracy of over 90% in individual maize segmentation from lidar data, which has a great potential to further improve the phenotype estimation accuracy at the individual plant level [36].

The non-destructive and high-accuracy characteristics made lidar technology an ideal tool in phenotyping applications. Especially, lidar technology is not influenced by light conditions, and therefore it can be used in field phenotyping practices. However, currently, the methods to acquire lidar data are still very limited [29]. Although the terrestrial lidar sensor can collect lidar point cloud with high accuracy and high point density, the data collection and preprocessing (e.g., registration among lidar scans) could be very time-consuming and complicate. Moreover, the fusion of lidar with other remote-sensing sensors (e.g., thermal sensor, solar-induced fluorescence sensor, and hyperspectral sensor) are needed to acquire physiology-related phenotypes beyond 3D structures [5, 27, 59, 60]. Therefore, a new platform that can automatically collect and register multi-source remote sensing data for high-throughput field-based phenotyping practices is in great need [29].

## Conclusion

This study used terrestrial lidar technology to extract temporal maize phenotypes. Overall, lidar showed a strong capability in estimating plant height and PLA non-destructively and accurately. Although the accuracy of PAI estimation from lidar was not as high as plant height and PLA estimations, it still reached a *R*^2^ of 0.70 and a RMSE of 0.15 m^2^/m^2^. Through the whole growth period, the three phenotypes of all 17 maize varieties showed a pattern of increasing first and then decreasing. In the heading and ripening stages, maize varieties with high drought tolerance tended to keep a low plant height and PAI without reducing PLA, which may help to both reduce the demand of water resources and ensure the photosynthesis rate. The relative low plant height and PAI at the tasseling stage would be useful indicators to identify maize varieties with high drought tolerance level during the growth period. Moreover, maize plants with high drought tolerance tended to keep lower upper level PAD than maize plants with low drought tolerance so that they could reduce the transpiration more efficiently.
